# Effect of dendritic cell-cytokine-induced killer cells in patients with advanced colorectal cancer combined with first-line treatment

**DOI:** 10.1186/s12957-017-1278-1

**Published:** 2017-11-28

**Authors:** Yunqing Xie, Lijie Huang, Luchuan Chen, Xiaowei Lin, Li Chen, Qiuhong Zheng

**Affiliations:** 1 Fujian Provincial Key Laboratory of Tumor Biotherapy, Fujian Medical University Cancer Hospital, Fuzhou, 350014 China; 2 Department of Abdominal Surgery, Fujian Medical University Cancer Hospital, Fuzhou, 350014 China

**Keywords:** Dendritic cells, Cytokine-induced killer cells, Adjuvant immunotherapy, Colorectal cancer

## Abstract

**Background:**

Surgical resection combined with adjuvant chemotherapy is considered as the gold-standard treatment for advanced colorectal cancer patients. These patients have a poor 5-year survival rate of 5% or less. Furthermore, a large dose of chemotherapy can produce adverse side effects and severe toxicity. Therefore, this retrospective study aimed to evaluate the efficacy of dendritic cell-cytokine-induced killer (DC-CIK) cell infusion as an adjuvant therapy in patients with advanced colorectal cancer combined with first-line treatment.

**Methods:**

A total of 142 patients with stage III/IV colorectal carcinoma who had been treated with first-line therapy were included in this study. Among these patients, 71 patients received first-line treatment only (non-DC-CIK group), while the other 71 patients who had similar demographic and clinical characteristics received a DC-CIK cell infusion combined with first-line treatment (DC-CIK group). These patients were followed up until August 2014. Data were analyzed by Kaplan-Meier and Cox regression.

**Results:**

Our results showed that the 5-year overall survival (OS) rate for the DC-CIK group versus the non-DC-CIK group was 41.3 versus 19.4% (*p* = 0.001) and the 5-year progression-free survival (PFS) rate for the DC-CIK group versus the non-DC-CIK group was 57.4 versus 33.6% (*p* = 0.022).

**Conclusions:**

Our results showed that patients with advanced colorectal cancer might benefit from DC-CIK immunotherapy combined with first-line therapy by significantly prolonging 5-year OS and PFS.

## Background

Colorectal cancer (CRC) is ranked the third leading malignancy and one of the leading causes of cancer-related deaths worldwide [1]. Over 20% of CRC patients have metastatic disease at the time of diagnosis [2]. In such advanced patients, who have a poor 5-year survival rate of 5% or less, surgical resection combined with adjuvant chemotherapy and/or radiotherapy has been recommended as the gold standard of treatment [3–7]. However, severe adverse side effects of such therapies limit the use of chemotherapy and radiotherapy in advanced CRC patients [8, 9]. In the past decade, cell-based immunotherapy has been reported to improve the clinical outcomes by altering tumor immune responses, improving prognosis and overall survival rates, and the quality of life in cancer patients [10–12]. Furthermore, it may minimize the presence of residual or resistant tumor cells after surgery, radiotherapy, and chemotherapy. However, studies of dendritic cell-cytokine-induced killer (DC-CIK) cells remain in preliminary stages, and the conclusion as to whether DC-CIK treatment in patients with distant metastatic disease is still poorly understood.

It has been previously reported that lymphocyte-activated killer (LAK) cells achieved clinical anti-tumor effects [13]. However, LAK cells have not been widely developed due to their inefficient proliferation and low anti-tumor activity. These hurdles were overcome by the identification of cytokine-induced killer (CIK) cells in 2005, which are rapidly proliferative and possess powerful anti-tumor activity and minimal toxicity [14]. CIK cells are a group of heterogeneous cells, characteristically expressing the T cell marker CD3 and natural killer (NK) cell markers CD56 or CD16. It has been demonstrated that the administration of CIK cells can improve overall survival rates and quality of life for malignant patients [15, 16]. In 2010, dendritic cells (DCs) were approved for treating metastatic prostate cancer by the FDA [17]. DCs are the most potent antigen-presenting cells regulating and maintaining T cell responses in order to provide protective anti-tumor immunity. Moreover, many studies have indicated that DCs may enhance the function of CIK by significantly improving proliferation and tumor-specific activity of CIK cells [18]. Indeed, the clinical benefits of DC-CIK have been reported in patients with solid tumors [14, 19, 20].

A few studies examining the effectiveness of DC-CIK/CIK immunotherapy for colorectal cancer have been reported [21, 22]. In the past studies, we have achieved promising findings demonstrating DC-CIK cell therapy as an adjuvant therapy for colorectal cancer and shown significant improvement in 3-year overall survival rate and disease-free survival rate [23, 24]. Wang et al. [25] showed DC-CIK immunotherapy significantly improved 3-year disease-free survival (DFS) in patients combined with chemotherapy. Gao et al. [21] also indicated that CRC are more sensitive to DC-CIK therapy than GC, and DFS and overall survival (OS) were both significantly prolonged in patients in the DC-CIK treatment group. However, few studies have focused on advanced CRC patients.

In the present study, we reviewed 142 advanced colorectal cancer treated with conventional or adjuvant DC-CIK therapy and analyzed their respective 1-, 3-, and 5-year OS rates and progression-free survival (PFS) rates. The purpose of our study was to investigate whether advanced colorectal cancer could benefit from DC-CIK combined with first-line treatments.

## Methods

### Patient selection

A retrospective analysis was carried out on the medical records of colorectal cancer patients in our hospital from January 1, 2006, to August 31, 2009. The inclusion criteria were as follows: (1) patients were pathologically diagnosed with colorectal cancer at stages III–IV according to the International Union Against Cancer’s (UICC 2002) classification based on pTNM system; (2) all patients received standard first-line treatment including surgical resection, adjuvant chemotherapy, and/or radiotherapy; and (3) another cohort of patients received at least two cycles of DC-CIK immunotherapy. Exclusion criteria were as follows: (1) patients who did not receive standard first-line treatment and (2) patients who were not in advanced stages (III–IV stages). After review, 142 patients who met the described criteria were enrolled in this study for further analysis (Table 1). Among them, 71 patients received DC-CIK treatment (DC-CIK group), whereas the other 71 patients without DC-CIK treatment were used as the control group for comparisons.

**Table 1:** Clinicopathological characteristics of 142 patients

Characteristics	Total (*n* = 142)	Control (*n* = 71)	DC-CIK (*n* = 71)	*p* value
Sex, male/female	79/63	40/31	39/32	0.44
Age (years), mean ± SD	55.4 ± 14.4	55.6 ± 14.3	55.3 ± 14.6	0.90
KPS, mean ± SD	70.4 ± 6.3	70.1 ± 5.7	70.6 ± 6.8	0.79
Adenocarcinoma				
Well differentiated	3	2	1	0.75
Moderately differentiated	104	54	50	
Poorly differentiated	22	9	13	
Unknown	13	6	7	
TNM stage (III/IV)	98/54	41/30	47/24	0.27
FOLFIRI chemotherapy cycles				
≤ 3	32	19	15	0.59
4–6	33	18	16	
> 6	44	21	24	
Radiotherapy (yes/no)	49/93	22/49	27/44	0.31

### First-line treatment

All enrolled CRC patients underwent primary tumor resection and postoperative FOLFIRI chemotherapy with or without radiotherapy. The FOLFIRI [irinotecan (CPT-11), leucovorin (LV), and 5-FU] regimen consisted of CPT-11 intravenous infusion (80 mg/m^2^) on day 1, intravenous LV infusion (400 mg/m^2^) on day 1, and intravenous bolus injection (0.4 g/m^2^) and 46-h infusion (2.4 g/m^2^) of 5-FU on day 1 and day 2. This regimen was repeated every 2 weeks.

### DC and CIK cell generation

Peripheral blood mononuclear cells (PBMCs) were separated from a 50-mL peripheral blood sample from each patient in the “DC-CIK” group by Ficoll-Paque density gradient centrifugation. PBMCs (1 × 10^7^ cells/mL) were plated onto six-well dishes (BD). Non-adherent cells were collected after 2 h of incubation at 37 °C under 5% CO_2_. Adherent cells were cultured in GT-T551 medium containing granulocyte-macrophage colony-stimulating factor (GM-CSF; 100 ng/mL; Amoytop) and interleukin-4 (IL-4; 50 ng/mL; Amoytop). The medium was replaced every 2 days. WT1 antigen (Miltenyi, UK) was then added to the medium at day 5 to a final concentration of 100 g/mL and cocultured with DCs for an additional 24 h.

Non-adherent cells were collected for CIK cell preparation. Cells were resuspended at a concentration of 5 × 10^6^ cells/mL with GT-T551 medium containing interferon-gamma (IFN-γ; 1000 U/mL; Novo protein) and cultured in 75-cm^2^ culture flasks at 37 °C under 5% CO_2_. Cells were treated with 1000 U/mL IL-2 (Sihuanpharm) and 50 ng/mL anti-CD3 monoclonal antibody (Takara) after 24 h. Cell density was readjusted to 5 × 10^6^ cells/mL by using fresh medium containing 500 U/mL IL-2 from day 2 to day 10. During the culture period, matured DCs were isolated by flow cytometry (Beckman) via DC-specific markers CD83, CD80, CD86, and HLA-DR (BD). Matured DCs were harvested and mixed with CIK cells at a ratio of 1:10 at day 7. On day 10, the co-cultured (DC-CIK) cells were examined by flow cytometry for CIK cell-specific markers with antibodies against CD3, CD8, and CD56 (Beckman).

### DC-CIK treatment and follow-up

DC-CIK cell-based treatment was combined with first-line treatments. Our study was approved by the Ethics Committee of Fujian Provincial Tumor Hospital (IRB-2003-01). All 71 patients received at least two cycles of DC-CIK cells and signed informed consent.

**Fig. 1:**
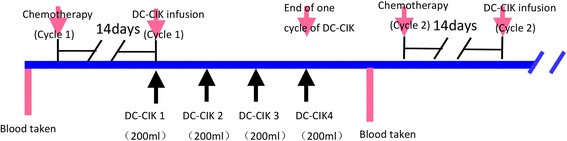
Illustration of the DC-CIK cell treatment protocol

Before transfusion, bacterial contamination and endotoxin levels were tested and were < 0.06 EU within 48 h. DC-CIK cells were then harvested and resuspended in normal salt solution and administered via intravenous infusion during intervals of chemotherapy twice a day for 4 days. The treatment protocol is shown in Fig. 1. Four transfusions were defined as one cycle. Patients received a total of 1–2 × 10^10^ cells at each cycle. The interval of every cycle was at least 2 weeks.

All patients were followed up after discharge, including clinic or telephone contact every 3 months for the first 2 years, 6 months for the next 3 years, and yearly thereafter from the fifth year. In this study, the follow-up deadline was August 2014. Follow-up included blood routine examination, colonoscopy every 6 months, CEA levels, and chest/abdominal CT scans every 6–12 months. In the DC-CIK group, lymphocyte subsets were determined before the start of every cycle of DC-CIK therapy. PET-CT was performed if tumor recurrence or metastases were suspected. Quality of life (QOL) of patients was evaluated according to the Karnofsky scores.

### Statistical analysis

SPSS 16.0 software was used for analyzing data. Data was showed in mean ± SD. The postoperative survival rate was compared with the Kaplan-Meier method. Clinically different characteristics between the DC-CIK group and the control group before and after DC-CIK therapy were compared by Student’s *t* test. The Cox proportional hazard regression test was used for multivariate analysis. *p* < 0.05 was set as statistical significance.

## Results

### Baseline characteristics of patients

A total of 142 patients with histologically confirmed colorectal carcinoma were enrolled in this study, 71 in the DC-CIK group and 71 in the control group. All patients were at stages III and IV. The clinical characteristics of these two groups are described in Table 1. There were no significant differences in demographic and clinical characteristics between these two groups. Despite DC-CIK transfusion, treatment strategies and medical management were similar in these two groups.

### Side effects of DC-CIK cell transfusion

During and after DC-CIK cell transfusion, six patients (8%) suffered from mild fever, chills, and fatigue; three also developed a headache; and one developed chest tightness and hypotension. All of them recovered after symptomatic treatment. There were no adverse reactions such as anaphylaxis. No patients presented with abnormal liver function tests (LFTs) or kidney function.

### Quality-of-life assessment

Quality of life in DC-CIK-treated patients had the KPS score of 76.48 ± 8.53 compared to that of the control of 67.74 ± 7.82, which is statistically significant (*p* < 0.05).

### Comparison of the effectiveness in clinic

In the median 76-month follow-up period of time, the median survival time of the patients in the DC-CIK group and non-DC-CIK group was 32 months (95%CI 18.89–45.41 months) and 17 months (95%CI 15.18–18.82 months), respectively, which was statistically significant (*p* < 0.001).

**Fig. 2:**
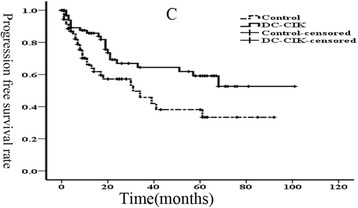
Kaplan-Meier estimate for progression-free survival (PFS) of patients

**Fig. 3:**
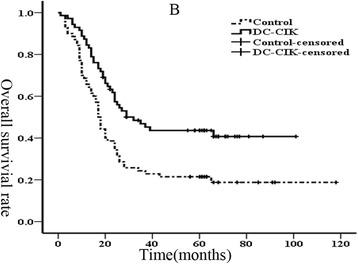
Kaplan-Meier estimate for overall survival (OS) of patients

We then subsequently analyzed the 1, 3, and 5-year progression-free survival rates, which were 85.3, 64.1, and 57.4%, respectively, in the DC-CIK group, whereas in the non-DC-CIK group were 65.0, 44.3, and 33.6%, respectively (*p* = 0.017; Fig. 2). Furthermore, the 1, 3, and 5-year overall survival rates were 84.5, 46.0, and 41.3%, respectively, in the DC-CIK group compared to 65.7, 23.4, and 19.4%, respectively, in the non-DC-CIK group (*p* = 0.001; Fig. 3). Our data indicated that DC-CIK cells as an adjuvant therapy combined with first-line treatment could significantly reduce mortality and recurrence for advanced colorectal carcinoma.

## Discussion

The present retrospective study of advanced CRC patients revealed several important findings. Firstly, DC-CIK cell adjuvant transfusion resulted in significantly prolonged 5-year OS rates compared to that of the control group. Secondly, the 5-year PFS rates were significantly improved. Finally, the 1- and 3-year OS and PFS rates in the DC-CIK group were higher than those in the control group. These results suggested that DC-CIK cell treatment, combined with first-line therapy, is an effective therapeutic strategy for treatment of advanced colorectal cancer.

DC-CIK cell treatment has been an established technique and is now widely used in cancer patients. Previous clinical studies have provided strong evidence supporting the efficacy of DC-CIK immunotherapy combined with chemotherapy and radiotherapy. Zhao et al. [26] showed that “the GP regimen combined with DC-CIK immunotherapy would reduce postoperative tumor recurrence and prolonging the survival time of patients with NSCLC.” Ma et al. [27] dedicated that “CIK cell therapy demonstrated a significant superiority in prolonging the median overall survival, PFS, DCR, ORR and QoL of HCC patients.” Other studies of gastric cancer [28], advanced renal cancer [29], and metastatic nasopharyngeal carcinoma [30] demonstrated that DC-CIK cell infusions could improve outcomes of patients. In our study, we selected 142 advanced CRC patients for evaluating the impact of adjuvant DC-CIK cell infusion, in particular in reference to 5-year OS and 5-year PFS rates, when combined with first-line routine therapy. Our results, consistent with previous studies in other advanced cancers, indicated that adjuvant DC-CIK cells could prolong 5-year OS and PFS rates for advanced CRC patients [26–30]. The 5-year OS rate of the control group in our study was similar to that in Gao et al.’s [21] showing 19.4 vs. 15% of effects, while Zhu et al. [31] suggested that CIK cells could prolong DFS but not OS rates for resected CRCs. Although the 3- and 5-year OS and PFS rates in our present DC-CIK group were lower than our other previously published studies in earlier staged CRC patients [23, 24], we have achieved promising results in advanced CRC patients and improved outcomes without adverse side effects. It was also shown that DC-CIK adjuvant transfusion therapy is safe and tolerated in advanced CRC patients.

Immunosuppression may be apparent in patients after surgery and chemotherapy, especially in advanced cancer patients. Moreover, DCs obtained from such patients may be dysfunctional and more likely to induce tolerance or non-productive T cell responses because of lost expression of antigens or MHC molecules in advanced patients [32]. Therefore, the clinical benefits of DC-CIK cells may be due to their strong anti-tumor activities through immumodulation [33]. DC-CIK cells not only eradicated residual tumor cells but also enhanced immune surveillance capacity of host cells by producing pro-inflammatory cytokines, such as IL-2 and IFN-γ to prevent or delay tumor recurrence [34, 35]. However, the responsible mechanisms for these clinical benefits remain to be elucidated.

While some objective clinical benefits have been demonstrated in patients with advanced or metastatic CRC, more studies are warranted. Firstly, due to the retrospective nature limitations, a well-designed prospective study should be carried out to examine these results further. Secondly, combined therapies may be optimized by comparison with other recent cell-based therapeutic studies. Indeed, it has been reported that chemotherapy and/or radiotherapy before cell transfusion can enhance anti-tumor effects in previous clinical studies [36]. Moreover, a recent study reported cytotoxic T lymphocyte-associated antigen 4 (CTLA-4) and programmed death ligand1 (PD-1) may play key roles in inhibiting T cell activation during cell therapies [37]. Currently, we are combining adoptive transfer of T cells with PD1 and CTLA-4 blockade after inductive chemotherapy and/or radiotherapy in a similar manner. In the future, we believe that several alternative modalities may improve immunotherapy with traditional therapies and ultimately change patient outcomes in advanced CRC cancer patients.

## Conclusion

In summary, DC-CIK cell adjuvant transfusion resulted in significantly prolonged 5-year OS rates compared to that of the control group. Secondly, the 5-year PFS rates were significantly improved; the 1- and 3-year OS and PFS rates in the DC-CIK group were higher than those in the control group. These results suggested that DC-CIK cell treatment, combined with first-line therapy, is an effective therapeutic strategy for treatment of advanced colorectal cancer.

## References

[1] Siegel R, Naishadham D, Jemal A (2013). Cancer statistics, 2013. CA Cancer J Clin.

[2] Cunningham D, Atkin W, Lenz HJ, Lynch HT, Minsky B, Nordlinger B (2010). Colorectal cancer. Lancet.

[3] Damin DC, Lazzaron AR (2014). Evolving treatment strategies for colorectal cancer: a critical review of current therapeutic options. World J Gastroenterol.

[4] Ke TW, Liao YM, Chiang HC, Chang SC, Wang PH, Chen YY (2016). Effectiveness of neoadjuvant concurrent chemoradiotherapy versus up-front proctectomy in clinical stage II–III rectal cancer: a population-based study. Asia Pac J Clin Oncol.

[5] Laurent M, Paillaud E, Tournigand C, Caillet P, Le Thuaut A, Lagrange JL (2014). Assessment of solid cancer treatment feasibility in older patients: a prospective cohort study. Oncologist.

[6] Sargent D, Sobrero A, Grothey A, O'Connell MJ, Buyse M, Andre T (2009). Evidence for cure by adjuvant therapy in colon cancer: observations based on individual patient data from 20,898 patients on 18 randomized trials. J Clin Oncol.

[7] Siegel R, Naishadham D, Jemal A (2012). Cancer statistics, 2012. CA Cancer J Clin.

[8] Sharif S, O'Connell MJ, Yothers G, Lopa S, Wolmark N (2008). FOLFOX and FLOX regimens for the adjuvant treatment of resected stage II and III colon cancer. Cancer Investig.

[9] Gallagher DJ, Kemeny N (2010). Metastatic colorectal cancer: from improved survival to potential cure. Oncology.

[10] Stroncek D, Berlyne D, Fox B, Gee A, Heimfeld S, Lindblad R (2010). Developments in clinical cell therapy. Cytotherapy.

[11] Popiela T, Kulig J, Czupryna A, Szczepanik AM, Zembala M (2004). Efficiency of adjuvant immunochemotherapy following curative resection in patients with locally advanced gastric cancer. Gastric Cancer.

[12] Avila MA, Berasain C, Sangro B, Prieto J (2006). New therapies for hepatocellular carcinoma. Oncogene.

[13] Oliver A, Canton L, Montanari M (1998). GM-CSF plus IL- 2 administration associated with multiple autologous LAK refusion can induce a major cytogenetic response in early relapsed CML after autologous transplantation: a case report. Bone Marrow Transplant.

[14] Hontscha C, Borck Y, Zhou H, Messmer D, Schmidt-Wolf IG (2011). Clinical trials on CIK cells: first report of the international registry on CIK cells (IRCC). J Cancer Res Clin Oncol.

[15] Iwai K, Soejima K, Kudoh S, Umezato Y, Kaneko T, Yoshimori K (2012). Extended survival observed in adoptive activated T lymphocyte immunotherapy for advanced lung cancer: results of a multicenter historical cohort study. Cancer Immunol Immunother.

[16] Tan J, Cang S, Ma Y, Petrillo RL, Liu D (2010). Novel histone deacetylase inhibitors in clinical trials as anti-cancer agents. J Hematol Oncol.

[17] Frohlich MW (2012). Sipuleucel-T for the treatment of advanced prostate cancer. Semin Oncol.

[18] Su X, Zhang L, Jin L, Ye J, Guan Z, Chen R (2010). Coculturing dendritic cells with zoledronate acid efficiently enhance the anti-tumor effects of cytokine-induced killer cells. J Clin Immunol.

[19] Chan JK, Hamilton CA, Cheung MK, Karimi M, Baker J, Gall JM (2006). Enhanced killing of primary ovarian cancer by retargeting autologous cytokine-induced killer cells with bispecific antibodies: a preclinical study. Clin Cancer Res.

[20] Li H, Wang C, Yu J, Cao S, Wei F, Zhang W (2009). Dendritic cell-activated cytokine-induced killer cells enhance the anti-tumor effect of chemotherapy on non-small cell lung cancer in patients after surgery. Cytotherapy.

[21] Gao D, Li C, Xie X, Zhao P, Wei X, Sun W (2014). Autologous tumor lysate-pulsed dendritic cell immunotherapy with cytokine-induced killer cells improves survival in gastric and colorectal cancer patients. PLoS One.

[22] Zhu H, Yang X, Li J, Ren Y, Zhang T, Zhang C (2014). Immune response, safety, and survival and quality of life outcomes for advanced colorectal cancer patients treated with dendritic cell vaccine and cytokine-induced killer cell therapy. Biomed Res Int.

[23] Wei ZQ, Yang JW, Chen LC, Zheng QH, Ying MG (2009). A retrospective analysis of postsurgical treatment for rectal cancer by chemotherapy and radiotherapy combined with dendritic cells. Fujian YikeDaxue Xuebao.

[24] Ying MG, Wei ZQ, Yang JW, Chen LC, Zheng QH (2010). Retrospective analysis of pos-operative chemo-radiotherapy combined with DC-CIK in the treatment of patients with colorectal cancer. ShiyongAizheng Zazhi.

[25] Wang ZX, Cao JX, Liu ZP, Cui YX, Li CY, Li D (2014). Combination of chemotherapy and immunotherapy for colon cancer in China: a meta-analysis. World J Gastroenterol.

[26] Zhao M, Li H, Li L, Zhang Y (2014). Effects of a gemcitabine plus platinum regimen combined with a dendritic cell-cytokine induced killer immunotherapy on recurrence and survival rate of non-small cell lung cancer patients. Exp Ther Med.

[27] Ma Y, Xu YC, Tang L, Zhang Z, Wang J, Wang HX (2012). Cytokine-induced killer (CIK) cell therapy for patients with hepatocellular carcinoma: efficacy and safety. Exp Hematol Oncol.

[28] Shi L, Zhou Q, Wu J, Ji M, Li G, Jiang J (2012). Efficacy of adjuvant immunotherapy with cytokine-induced killer cells in patients with locally advanced gastric cancer. Cancer Immunol Immunother.

[29] Wang D, Zhang B, Gao H, Ding G, Wu Q, Zhang J (2014). Clinical research of genetically modified dendritic cells in combination with cytokine-induced killer cell treatment in advanced renal cancer. BMC Cancer.

[30] Li JJ, MF G, Pan K, Liu LZ, Zhang H, Shen WX (2012). Autologous cytokine-induced killer cell transfusion in combination with gemcitabine plus cisplatin regimen chemotherapy for metastatic nasopharyngeal carcinoma. J Immunother.

[31] Zhu Y, Zhang H, Li Y, Bai J, Liu L, Liu Y (2013). Efficacy of postoperative adjuvant transfusion of cytokine-induced killer cells combined with chemotherapy in patients with colorectal cancer. Cancer Immunol Immunother.

[32] Kiessling R, Wasserman K, Horiguchi S, Kono K, Sjoberg J, Pisa P (1999). Tumor-induced immune dysfunction. Cancer Immunol Immunother.

[33] Swartz MA (2014). Immunomodulatory roles of lymphatic vessels in cancer progression. Cancer Immunol Res.

[34] Schlom J (2012). Recent advances in therapeutic cancer vaccines. Cancer Biother Radiopharm.

[35] Jiang J, Wu C, Lu B (2013). Cytokine-induced killer cells promote antitumor immunity. J Transl Med.

[36] Dudley ME, Yang JC, Sherry R, Hughes MS, Royal R, Kammula U (2008). Adoptive cell therapy for patients with metastatic melanoma: evaluation of intensive myeloablative chemoradiation preparative regimens. J Clin Oncol.

[37] Drake CG (2012). Combination immunotherapy approaches. Ann Oncol.

